# CRISPR/Cas9 Technology for Enhancing Desirable Traits of Fish Species in Aquaculture

**DOI:** 10.3390/ijms25179299

**Published:** 2024-08-27

**Authors:** Minli Zhu, Sahr Lamin Sumana, Mukhtar Muhammad Abdullateef, Opeoluwa Christiana Falayi, Yan Shui, Chengfeng Zhang, Jian Zhu, Shengyan Su

**Affiliations:** 1 Wuxi Fisheries College, Nanjing Agricultural University, Wuxi 214081, China; zhuminli1023@163.com (M.Z.); sl5284sumana@gmail.com (S.L.S.); mukmuha1989@gmail.com (M.M.A.); opeoluwafalayi@gmail.com (O.C.F.); 2 National Demonstration Center for Experimental Fisheries Science Education, Shanghai Ocean University, Shanghai 201306, China; 3 Key Laboratory of Integrated Rice-Fish Farming Ecology, Ministry of Agriculture and Rural Affairs, Freshwater Fisheries Research Center, Chinese Academy of Fishery Sciences, Wuxi 214081, China; shuiy@ffrc.cn (Y.S.); zhangcf@ffrc.cn (C.Z.); zhuj@ffrc.cn (J.Z.)

**Keywords:** CRISPR/Cas9, desirable fish traits, aquaculture, public perceptions, environmental consideration

## Abstract

Aquaculture, the world’s fastest-growing food production sector, is critical for addressing food security concerns because of its potential to deliver high-quality, nutrient-rich supplies by 2050. This review assesses the effectiveness of CRISPR/Cas9 genome editing technology in enhancing desirable traits in fish species, including growth rates, muscle quality, disease resistance, pigmentation, and more. It also focuses on the potential effectiveness of the technology in allowing precise and targeted modifications of fish DNA to improve desirable characteristics. Many studies have reported successful applications of CRISPR/Cas9, such as knocking out reproductive genes to control reproduction and sex determination, enhancing feed conversion efficiency, and reducing off-target effects. Additionally, this technology has contributed to environmental sustainability by reducing nitrogen-rich waste and improving the nutritional composition of fish. However, the acceptance of CRISPR/Cas9 modified fish by the public and consumers is hindered by concerns regarding public perception, potential ecological impacts, and regulatory frameworks. To gain public approval and consumer confidence, clear communication about the editing process, as well as data on the safety and environmental considerations of genetically modified fish, are essential. This review paper discusses these challenges, provides possible solutions, and recommends future research on the integration of CRISPR/Cas9 into sustainable aquaculture practices, focusing on the responsible management of genetically modified fish to enable the creation of growth and disease-resistant strains. In conclusion, this review highlights the transformative potential of CRISPR/Cas9 technology in improving fish traits, while also considering the challenges and ethical considerations associated with sustainable and responsible practices in aquaculture.

## 1. Introduction

Aquaculture is the fastest-growing sector in food production worldwide, addressing challenges related to global food security caused by population growth, climate change, and limited resources [1]. The projected global population of 9.7 billion by 2050 will require a 25 to 70 percent increase in food production. However, in order to meet these global challenges, it is crucial to have high-quality, nutrient-rich food sources, which is where aquaculture comes in. In 2011, aquaculture was estimated to account for 85–89% of global production for household consumption, with 51% and 52%, respectively, representing the total population and undernourished population [2]. From the 1990s to 2022, human consumption of aquaculture and fisheries products has doubled from 81.6 to 164.6 million metric tonnes annually [3]. Aquaculture also plays a significant role in the food supply, accounting for over 57% of aquatic animal foods in 2022 [3]. The long-term growth and expansion of the aquaculture industry depend on protein-rich food products to meet the needs of the growing global population. There are concerns about the rising demand for aquatic products due to population growth, and the aquaculture sector seems to offer a promising solution. This has been made possible through the relentless efforts of researchers in advancing genetic engineering, particularly through the use of CRISPR/Cas9 technology to address disease outbreaks, slow growth rates, and environmental stressors in various fish species.

CRISPR/Cas9 is a groundbreaking genetic engineering tool that allows for precise and targeted modifications of fish DNA to enhance desirable traits such as color pigmentation, growth, muscle quality, and disease resistance [4,5,6]. This technology surpasses traditional breeding techniques, offering a cheaper, easier, and more precise method for genetic improvement [7,8,9,10]. It enables precise genome editing to improve essential traits, including growth performance (such as increased body weight, length, and muscle fiber development), muscle quality, disease resistance, and sex determination [11,12,13,14,15,16,17]. Additionally, CRISPR/Cas9 technology offers a promising solution for enhancing disease resistance in fish by targeting immune-related genes or pathogen recognition pathways, thereby reducing reliance on antibiotics and chemical treatments [7]. Moreover, this technology has revolutionized modern aquaculture by genetically enhancing key characteristics of fish species. For example, researchers have successfully eliminated germ cells responsible for reproductive cell sex differentiation in Atlantic salmon [18], improved feed conversion efficiency for accelerated growth rates in yellow catfish [13], achieved efficient gene mutations in tilapia, and minimized off-target effects [19].

The environment is vital for the sustainability of the aquaculture industry. This technology not only enhances desirable traits in fish but also addresses environmental sustainability and consumer preferences. Some authors have modified fish to produce less nitrogen-rich waste, reducing the environmental impact of aquaculture. They have also improved the nutritional composition of fish, increasing omega-3 fatty acid levels and reducing undesirable compounds. The fatty acid desaturase *fads2* gene in rainbow trout has been edited to increase the production of omega-3 fatty acids, which are highly valued by consumers.

However, the acceptance of this technology by the public and consumers is hindered by factors such as public perception and potential ecological impacts [20] raised ethical concerns about genetic engineering in animals, particularly through gene knockout and knock-in methods. The commercialization of CRISPR/Cas9 products requires risk analysis, regulatory approval, and public acceptance. In addition, public approval and consumer confidence in genetically modified fish are influenced by the reliability of health, safety, and environmental considerations [21]. To gain public approval and consumer confidence, it is crucial to provide clear explanations about the process of editing fish traits, as well as regulatory frameworks and data on genetically modified fish (GMF) to address these concerns.

CRISPR/Cas9 technology has garnered interest in the field of aquaculture in recent years. However, there is currently a lack of a comprehensive review that encompasses its various applications and the challenges it poses in modifying key desirable traits in fish. Previous studies have primarily concentrated on explaining the principles of the technology, its advantages, and its utilization in enhancing economically significant traits [22].

Therefore, this review paper evaluates the effectiveness of CRISPR/Cas9 technology in improving traits in aquaculture fish species, including growth rates, muscle quality, disease resistance, and environmental stress tolerance. It also discusses the challenges and ethical considerations involved in sustainable and responsible practices.

## 2. Major Fish Species Traits Improved by Genome Editing in Aquaculture

Aquaculture is vital for global food security and providing dietary protein. China leads in genetic enhancement, particularly using the CRISPR-Cas9 system, to improve carp strains. This technology has revolutionized desirable traits in aquaculture, particularly in developing countries.

CRISPR-Cas9 is used to target and modify specific genes in fish, leading to a range of improvements. For example, this technology has been utilized to alter pigmentation pathways, resulting in enhanced color changes in ornamental species like goldfish and common carp [23,24]. Additionally, CRISPR-Cas9 has been applied to increase growth rates in economically important species such as tilapia and common carp, contributing to more efficient production systems [25,26]. It is essential that the area of improvement be disease resistance, as genetic modifications have enhanced the immune response in species such as Atlantic salmon and rainbow trout, reducing mortality from common aquaculture pathogens [27]. In medaka, CRISPR-Cas9 has improved feed conversion efficiency, helping to lower production costs and minimize environmental impact [28].

The nutritional quality of farmed fish has also seen improvements through genome editing. For instance, modifications in Atlantic salmon have enhanced omega-3 fatty acid metabolism, increasing their nutritional value for human consumption [29]. Furthermore, CRISPR-Cas9 has been used to address reproductive challenges by controlling sterility and fertility in Nile tilapia and Atlantic salmon, aiding in better population management [30,31,32,33].

Moreover, sex control through genome editing has allowed for the manipulation of sex-determination genes in species like Nile tilapia and rainbow trout, optimizing production efficiency by controlling sex ratios [29]. The technology has also improved tolerance to abiotic stresses, such as low oxygen levels and increased salinity in Atlantic salmon, enhancing survival rates in challenging environments [27].

**Figure 1:**
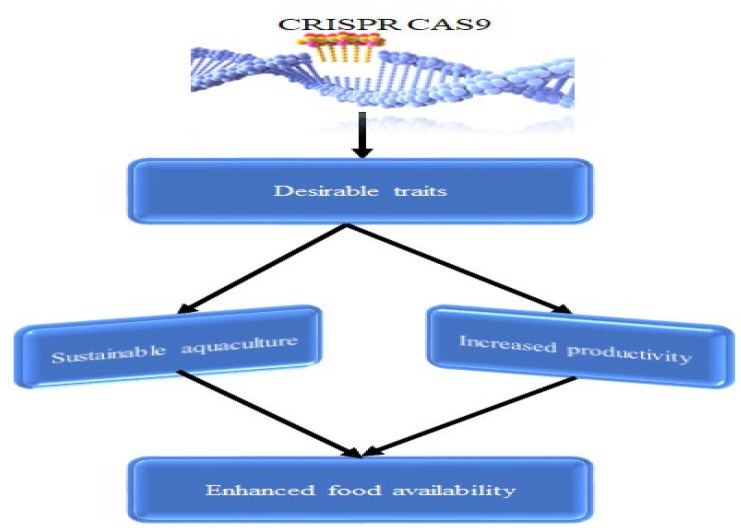
The conceptual framework outlines how the CRISPR-Cas9 model can be applied to enhance desirable traits in fish, with the ultimate goal of improving food availability.

Additionally, several researchers have utilized CRISPR-Cas9 to edit immune genes in different fish species, further enhancing disease resistance and overall health [34,35,36]. These advancements in genome editing have significantly contributed to reducing undesirable traits while enhancing desirable ones, making aquaculture more efficient and sustainable. With CRISPR-Cas9 technology, the aquaculture industry can continue to evolve and meet the growing global demand for nutritious and sustainably produced protein food (Figure 1).

## 3. Disease Resistance

Disease resistance refers to a fish species’ ability to tolerate internal and external factors that can disrupt its physiological or morphological functions. These factors include infectious parasites, poor water quality (pollution), and climate change. Potts [14] provided a helpful definition, stating that disease resistance is a broad term that covers a spectrum ranging from increased tolerance to complete protection.

Disease poses a significant challenge in aquaculture development and negatively impacts overall production yield, reducing fish farmers’ profits. This situation has prompted genetic improvements in disease-resistant fish species, leading to new approaches for mapping loci that affect disease resistance. Studies have shown the potential of marker-assisted selection, particularly genomic selection, in accelerating genetic gain in target traits [37].

CRISPR/Cas9-mediated genome editing has emerged as a powerful tool to enhance disease resistance in fish. By integrating vector-engineered antimicrobial peptide genes (*AMGs*), CRISPR/Cas9 can decrease bacterial colony-forming units in fish tissues, increase post-infection survival rates, and alter the expression of immune-related genes [38]. This technology allows for precise modifications in the fish genome, improving disease resistance, growth, and reproduction [39].

One successful application of CRISPR/Cas9 is in salmon, where it has improved resistance to infections such as infectious pancreatic necrosis (*IPN*), bacterial cold-water disease, and viral infections. In grass carp cells, researchers have used CRISPR/Cas9 technology to knock out the *JAM-A* gene, which plays a role in viral entry into host cells, conferring resistance against grass carp reovirus (*GCRV*) infection [39].

Furthermore, CRISPR/Cas9-based transgenesis allows for the site-directed knock-in of foreign genes at multiple loci, enhancing disease resistance in combination with other desirable traits such as fast growth and enriched fatty acid content [40]. For instance, this approach has been used to integrate genes that confer resistance to specific pathogens while simultaneously improving growth rates and nutritional quality in fish species such as tilapia and catfish [41].

In tilapia, CRISPR/Cas9 has been employed to edit genes involved in the immune response, leading to enhanced resistance against bacterial pathogens like *Streptococcus agalactiae* and *Aeromonas hydrophila* [42]. Similarly, in catfish, CRISPR/Cas9 has been used to target genes that regulate immune pathways, resulting in increased survival rates following pathogen exposure [37,43].

In conclusion, CRISPR/Cas9 holds significant promise for modulating the innate immune systems of fish and combating various pathogens. It is a valuable tool in aquaculture disease management, offering a path toward sustainable and resilient aquaculture systems [37,44].

## 4. Fish Growth and Muscle Quality

CRISPR/Cas9-based genome editing has been extensively applied in various fish species, particularly in freshwater fish used in aquaculture. This technology has been used in species such as Nile tilapia [45], channel catfish [12], southern catfish, common carp [46], rohu [47], grass carp [39], and rainbow trout.

Some of these genome-edited fish have demonstrated superior traits, such as improved disease resistance or enhanced growth performance. For example, myostatin-knockout channel catfish exhibited a 29.7% increase in mean body weight compared to control individuals [12].

Fish growth and muscle quality are important traits for the development of aquaculture worldwide. Advances in genetic modification and biotechnology have allowed researchers to target specific genes to improve these traits in different fish species. Numerous studies have successfully modified genes associated with growth and muscle development, producing promising results in species such as red sea bream, channel catfish, and common carp [26].

One significant area of research focuses on manipulating growth hormone-related genes. For example, alterations to the myostatin (*mstn*) gene, which negatively regulates muscle growth, have been shown to enhance muscle growth and mass in various fish species. In blunt snout bream, disrupting the *mstn* gene resulted in increased muscle mass and body weight [48], while similar modifications in olive flounder led to significant muscle mass enhancement [49]. Additionally, gene editing of *mstn* in Red Sea bream has improved skeletal muscle mass and reduced body length, optimizing fish size for commercial purposes [50].

In common carp, targeted genetic interventions have improved growth performance, demonstrating the potential of genetic approaches to enhance aquaculture efficiency [51]. Beyond traditional genetic modification, CRISPR/Cas9 technology has emerged as a powerful tool for precise genome editing. In zebrafish, CRISPR has been used to identify genes related to the enteric nervous system, including those encoding opioid receptors, which play a vital role in gut development [52].

CRISPR/Cas9 technology has also facilitated the study of developmental processes and human diseases by allowing researchers to create gene knockouts and observe phenotypic outcomes in model organisms like zebrafish [53]. In aquaculture, this technology holds great promise for improving fish growth, muscle quality, animal welfare, and farming efficiency [54].

Moreover, transgenic approaches have been used to overexpress growth hormone genes in species like Atlantic salmon, resulting in rapid growth rates and increased biomass production [55]. Such genetic enhancements are crucial for meeting the growing global demand for fish protein.

In addition to genetic modification, other factors such as nutrition, environmental conditions, and selective breeding play important roles in optimizing fish growth and muscle quality. Studies have shown that dietary supplements, such as amino acids and omega-3 fatty acids, can improve muscle texture and nutritional value in aquaculture species [56,57].

## 5. Off-Target Effects in CRISPR/Cas9 and Advances in Aquaculture

CRISPR/Cas9 technology can introduce off-target effects, affecting genomes in non-target locations and potentially causing adverse effects on organisms, including fish species. However, recent advancements have led to the development of high-fidelity Cas9 variants that significantly reduce off-target activity. For example, the SpCas9-HF1 and eSpCas9 variants have shown reduced non-specific binding and cleavage compared to the wild-type Cas9, thereby improving target specificity in fish species [58]. Additionally, the optimization of guide RNA (gRNA) design has played a critical role in minimizing off-target effects. Algorithms that predict potential off-target sites based on sequence similarity and binding affinity have become more sophisticated. Tools like CRISPR-DO, CCTop, and others assist researchers in designing more specific gRNAs [59].

Furthermore, new technologies such as base editors and prime editors offer precision without causing double-strand breaks, thus further reducing the risk of off-target mutations. Base editing allows for targeted nucleotide changes without introducing double-strand breaks, while prime editing enables more precise insertions, deletions, or corrections [60,61]. In addition, advancements in delivery systems, including nanoparticles and viral vectors, have improved the precision of CRISPR/Cas9 by enhancing gene editing accuracy at the cellular level. These systems ensure more effective delivery of CRISPR/Cas9 components to target cells while minimizing exposure to off-target sites [62,63].

Zebrafish are widely used as a model organism in CRISPR research due to their transparent embryos and rapid development. Successful applications of CRISPR/Cas9 in zebrafish have demonstrated its effectiveness in gene knockout, gene knock-in, and gene modification while minimizing off-target effects through the use of high-fidelity Cas9 variants and improved gRNA design [64] (see Figure 1).

**Figure 2:**
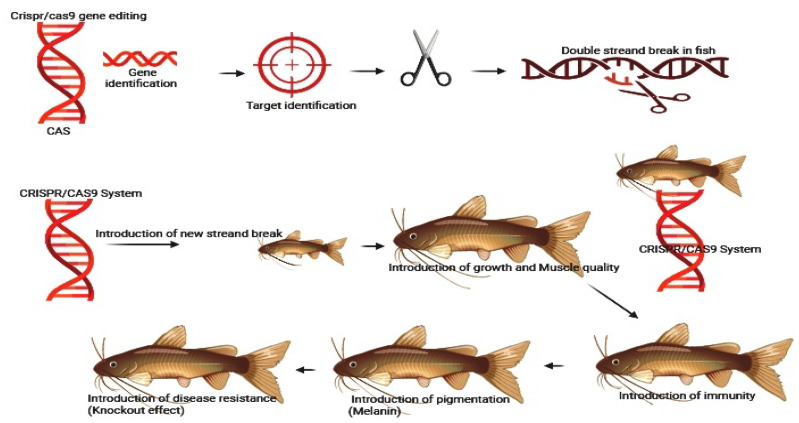
The application of CRISPR/Cas9 in aquaculture involves several steps. First, a specific gRNA is designed to match the target gene sequence. Then, the Cas9 protein binds to the target DNA, causing a double-strand break. Finally, the break is repaired.

In tilapia, CRISPR/Cas9 has been employed to enhance traits such as growth rate and disease resistance. Recent studies have utilized improved Cas9 variants and optimized gRNAs to achieve precise genetic modifications with reduced off-target effects [25,65]. CRISPR/Cas9 technology has also been applied to Atlantic salmon to improve traits such as growth and disease resistance. Research in this area has focused on utilizing advanced Cas9 variants and delivery methods to achieve precise editing with minimal off-target mutations [22]. The mechanism used by CRISPR/Cas9 in knockout genes in different fish species are indicated in Figure 2.

## 6. Sex Determination

The process of differentiating between male or female in sexually reproducing organisms is called sex determination. Sex determination in fish is a complex process that involves genetic, environmental, and epigenetic factors [59]. CRISPR/Cas9 technology has significantly contributed to the understanding of sex determination and differentiation in fish by using various genetic and environmental signals [16,66]. Fish, being the largest vertebrates, are ideal for studying sex determination and differentiation due to their diverse aquatic habitats, reproductive techniques, and sex characteristics. The gene-editing technology, CRISPR/Cas9, has revolutionized research on fish genetic traits by enabling precise DNA alteration and opening up diverse applications.

Sex determination and differentiation in fish involve a balance between male-promoting and female-promoting factors in somatic cells. Mutations in genes that favor females can decrease estrogen and increase androgen production, leading to female-to-male sex reversal [67,68,69,70,71]. Conversely, mutations in genes that favor males can increase estrogen (E2) production and decrease ketotestosterone (*11-KT*) production, resulting in male-to-female sex reversal [71].

CRISPR/Cas9 has been used in several studies to investigate sex determination genes in fish by disrupting these genes and observing the resulting phenotypes. In *Nile tilapia*, the anti-Müllerian hormone (amh) gene, which is involved in male sex determination, was targeted using CRISPR/Cas9. Disrupting the amh gene led to the development of phenotypic females, even among genetic males, demonstrating the gene’s significance in tilapia sex determination [45,72].

Similarly, in medaka fish, the *dmrt1* gene, a key regulator of male sex determination in this species, was targeted using CRISPR/Cas9. Disrupting *dmrt1* resulted in phenotypic females developing from genetic males, highlighting the gene’s crucial role in medaka sex determination [25,72].

In zebrafish, which have a polygenic sex determination system, CRISPR/Cas9 has been utilized to investigate the roles of various candidate genes, such as *dmrt1*, *sox9a*, and *amhy*. These studies have provided insight into the complex genetic architecture of sex determination in zebrafish [73].

Furthermore, CRISPR/Cas9 has been employed to discover new sex determination genes. For example, a genome-wide CRISPR/Cas9 screening in rainbow trout identified the *sdY* gene as the master regulator of male sex determination in this species [74].

## 7. Effects of CRISPR/Cas9 Technology on Different Biological and Environmental Aspects

The modern fish farming system faces several discouraging factors that result in low production and impact sustainability. These factors include disease outbreaks, poor growth rates, and environmental degradation [22,75,76]. Conventional breeding methods have had limited success in addressing these issues. However, the emergence of CRISPR/Cas9 technology offers new possibilities for improving the sustainability of aquaculture.

CRISPR/Cas9, initially designed for precise genetic modification, has revolutionized genome study and manipulation, enabling researchers to create genetically modified organisms with desired traits [4], alter sex determination [45], model human diseases in animal systems [77], and conduct high-throughput genetic screens to uncover gene functions [78]. This gene-editing technology, introduced as a revolutionary tool, has significantly impacted various biological and environmental domains [79]. Its versatility and precision have seen it expanded to various applications, including controlling invasive species, engineering microorganisms for bioremediation and other environmental cleanup efforts, and developing genetically modified fish for sustainable aquaculture [22].

Genome editing, particularly with the CRISPR/Cas9 technique, is being explored as a potential solution to the challenges faced by the aquaculture industry. The development of more effective and affordable genome editing techniques has made this approach increasingly viable. For example, the AquAdvantage salmon was developed in Canada in 2016 and approved for food production in the U.S. in 2019 [80]. This genetically modified salmon was created by combining the growth hormone gene from Chinook salmon with a promoter sequence from the antifreeze protein gene of an ocean pout, creating a gene construct that was then inserted into an Atlantic salmon egg to produce AquAdvantage salmon [81]. Similarly, Nile tilapia was genetically modified in Argentina in 2018 to enhance growth rates and disease resistance [82]. Table 1 provides a summary of the traits most commonly targeted for genome editing in fish aquaculture [83].

## 8. Effects of Using CRISPR/Cas9 in Gene Editing on Different Fish Species

The CRISPR/Cas9 gene editing technology has shown significant potential for improving various traits in fish and aquaculture species. Researchers have used this powerful tool to gain important insights into the genetic regulation of key physiological and production-related characteristics. CRISPR/Cas9 has aided researchers in developing gene drives, which are genetic engineering technologies that enhance the chances of a gene being inherited by the next generation. This allows for the more rapid spread of specific traits throughout a population compared to traditional inheritance methods. Gene drives achieve this by ensuring that the engineered gene is inherited at significantly higher frequencies, reaching close to 100%, instead of the typical 50% inheritance rate.

A notable example is the work of Li et al. [25], who used CRISPR/Cas9 to create sterile, all-male populations of Nile tilapia. Their study found that these genetically modified tilapia showed significantly improved growth rates compared to normal mixed-sex populations. Additionally, producing sterile fish helps mitigate the environmental risks associated with escaped farm-raised tilapia, which can disrupt local ecosystems through competition and interbreeding with wild populations. This study has been influential in demonstrating the potential of CRISPR technology to enhance aquaculture productivity while minimizing ecological impacts.

Similarly, Wargelius et al. [18] applied CRISPR/Cas9 gene editing to Atlantic salmon, conferring resistance against significant viral pathogens such as infectious pancreatic necrosis virus (IPNV) and salmon alphavirus (SAV). By disrupting or modifying host genes that these viruses rely on for infection and replication, the researchers were able to produce salmon lines with increased disease resistance. This advancement is significant, as viral diseases can cause substantial losses in Atlantic salmon aquaculture.

Building on these successes, other studies have used CRISPR/Cas9 to engineer disease resistance in various commercially important fish species. Godel (2015) reported developing carp resistant to koi herpesvirus (KHV), a devastating pathogen that can decimate carp populations. Ma et al. and Chakrapani et al. [39,47] have also explored CRISPR-based approaches to enhance disease resistance in grass carp and common carp, respectively.

In addition to disease resistance, researchers have used CRISPR/Cas9 to modify growth-related genes in several fish species. Zhong et al. [46] targeted myostatin, a negative regulator of muscle growth, in common carp, resulting in increased body size and growth rates. Similar studies have been conducted in channel catfish [12], tiger pufferfish, red sea bream [50], and olive flounder [49], demonstrating the versatility of this technology in optimizing commercially valuable production traits.

CRISPR/Cas9 has also enabled the development of novel phenotypes in aquatic organisms beyond these production-focused applications. Segev-Hadar et al. [91] used the technology to create true albino Nile tilapia with distinctive pink eyes, while Yu et al. [19] modified the *myostatin* gene in Pacific oysters, resulting in altered growth and muscle development. Furthermore, Gui et al. [92] reported successful genome editing of the ridgetail shrimp, showcasing the broad applicability of CRISPR/Cas9 across diverse aquatic species (Table 2).

## 9. Potential Socioeconomic Impacts of Widespread Adoption of CRISPR-Based Disease-Resistant Aquaculture in Developing Regions

The rapid progress of gene-editing technologies like CRISPR/Cas9 has created new opportunities for the aquaculture industry in developing regions. Disease-resistant aquaculture using CRISPR/Cas9 has the potential to greatly affect the availability, cost, and nutritional quality of seafood, as well as the livelihoods of small-scale and subsistence-level fish farmers [94]. However, the widespread use of this technology also raises important socioeconomic issues that require careful examination.

Improving disease resistance and productivity in aquaculture species through advancements in this technology can significantly increase the availability and affordability of nutritious seafood. This has the potential to enhance food security and nutritional outcomes, particularly for vulnerable populations [95]. These improvements could also boost incomes for small-scale and subsistence-level fish farmers, helping to lift them out of poverty and enhance their economic resilience [94]. Furthermore, implementing CRISPR/Cas9-based disease-resistant aquaculture could strengthen the overall industry in developing regions, contributing to economic development and integration into global aquaculture markets.

However, several potential challenges must be addressed. The high initial costs and intellectual property restrictions associated with CRISPR/Cas9 technology may limit access for small-scale and resource-constrained producers, potentially worsening existing inequalities [96]. To ensure equitable access, careful policies and support mechanisms are needed to prevent the marginalization of vulnerable groups. The introduction of CRISPR/Cas9-based aquaculture could disrupt traditional fishing and aquaculture practices, potentially displacing communities that rely on these activities for their livelihoods [94]. Inclusive stakeholder engagement and transition support are essential to mitigate these challenges and ensure a just and sustainable transformation.

Additionally, the widespread adoption of CRISPR/Cas9-modified aquaculture species could pose unforeseen ecological risks, such as disrupting local ecosystems or introducing invasive species [96]. Robust environmental impact assessments and appropriate safeguards are necessary to minimize these risks. The introduction of novel biotechnologies including CRISPR/Cas9 in aquaculture may also face resistance or skepticism from local communities due to cultural, religious, or traditional beliefs.

## 10. CRISPR-Based Aquaculture on Global Aquatic Product Trade and Food Security in Developing Regions

The successful adoption of CRISPR-based disease-resistant aquaculture has the potential to significantly increase the overall production and supply of certain aquatic species in global markets [94]. Regions with access to CRISPR technology may gain a competitive advantage in the global aquatic product trade, potentially outcompeting other producers and altering the relative competitiveness of different aquaculture-producing countries. Additionally, the introduction of CRISPR-based aquaculture technologies may lead to new trade barriers, such as regulations or tariffs, aimed at protecting domestic aquatic product industries in certain countries.

However, the patenting and licensing of CRISPR-based aquaculture technologies could result in increased market concentration, with a few multinational corporations or research institutions controlling the supply and distribution of these modified aquatic species [96]). This concentration of power could have far-reaching implications for global seafood trade, impacting prices, accessibility, and the bargaining power of small-scale producers in developing regions.

On the positive side, enhanced productivity and reduced disease-related losses associated with CRISPR-based aquaculture can make aquatic products more affordable and accessible in developing regions, thereby contributing to improved food security and nutrition [95]). Nevertheless, the introduction of CRISPR-modified aquaculture species presents both benefits and challenges for vulnerable populations reliant on aquatic products for essential nutrients.

The widespread implementation of this technology may disrupt traditional fishing practices and local food systems in developing regions, leading to unintended consequences such as the displacement of small-scale producers and the erosion of local food cultures [94]). Moreover, the high costs and intellectual property barriers associated with CRISPR technology may hinder access to disease-resistant aquaculture species for resource-constrained producers and consumers in developing regions [96]. To address these issues, careful policy interventions and support mechanisms are essential to ensure equitable access and prevent the marginalization of vulnerable groups, including small-scale and subsistence aquaculture producers, indigenous and local communities, and women and marginalized genders.

The large-scale adoption of CRISPR-modified aquaculture species could also have ecological repercussions, such as disrupting local ecosystems and introducing invasive species [96]. Robust environmental impact assessments and appropriate safeguards are necessary to mitigate these risks.

Governments and international development organizations can help address these challenges by providing targeted subsidies and financial assistance to small-scale producers and marginalized communities in developing regions. These subsidies can cover the costs of CRISPR-based aquaculture technology acquisition, implementation, and capacity building, enabling these resource-constrained groups to benefit from the productivity and disease resistance offered by CRISPR-modified aquaculture species.

Policymakers can also work to establish fair and accessible licensing schemes for CRISPR-based aquaculture technologies. This could involve negotiating reasonable royalty rates, tiered pricing structures, or even compulsory licensing arrangements to ensure that producers in developing regions can affordably access these transformative innovations.

Additionally, governments and international organizations can facilitate the transfer of CRISPR expertise and technical knowledge to research institutions and extension services in developing regions. This can be achieved through training programs, joint research collaborations, and the establishment of technology demonstration centers to build local capacity and empower small-scale producers to effectively utilize CRISPR-based aquaculture technologies.

Comprehensive regulatory frameworks should be developed to ensure the safe and responsible use of CRISPR-modified aquaculture species, including robust environmental impact assessments and biosafety measures. These regulations should balance the need for technological advancement with the protection of local ecosystems and traditional food systems in developing regions.

Finally, policymakers and development agencies should actively engage with local communities, small-scale producers, and other key stakeholders to understand their needs, concerns, and priorities. This inclusive approach will help ensure that policy interventions and support mechanisms are tailored to the specific contexts and vulnerabilities of marginalized populations, promoting equitable access to CRISPR technology in aquaculture.

## 11. Mitigation Strategies for the Sustainable Application of CRISPR/Cas9 in Aquaculture: Identifying Environmental and Ecological Impacts

The application of CRISPR/Cas9 technology in aquaculture shows promise for improving desirable traits in fish, such as growth rates, disease resistance, and stress tolerance. However, there are concerns about potential environmental and ecological impacts. One major concern is the possibility of gene flow from genetically modified fish to wild populations, which could lead to genetic pollution, reduced genetic diversity, and instability in ecosystems. Gene flow has the potential to disrupt aquatic ecosystems and have unforeseen ecological effects. CRISPR/Cas9 can also result in unintended off-target effects, where genes other than the intended targets are modified, potentially causing unpredictable outcomes [97]. To prevent unintended ecological consequences, it is crucial to assess off-target effects and understand broader ecological interactions. Off-target effects can result in unintended genetic mutations that disrupt gene function and impact cellular processes. In ecological contexts, these effects can change the competition among fish species and the dissemination of modified traits. Moreover, off-target effects give rise to safety concerns in medical applications.

Effective containment strategies are essential for minimizing the risk of transgenic fish escaping and causing gene flow to wild populations. Studies have demonstrated that implementing rigorous physical containment measures, such as multiple barriers, can significantly reduce the likelihood of transgenic fish escape. In fact, some strategies have been shown to decrease the risk by up to 99.9% [98]. Genetic strategies, such as Genetic Use Restriction Technologies (GURTs), can limit the spread of transgenes by approximately 99% [97]. These technologies aim to prevent genetically modified organisms from surviving and reproducing in the wild. By implementing multiple, independent, and redundant genetic safeguards, the probability of functional transgene escape can be reduced to less than one in a million [99].

Environmental risk assessments are crucial for evaluating potential adverse impacts. They can mitigate up to 80% of adverse environmental effects [100]. Incorporating ecological modeling and field trials can improve prediction accuracy by up to 90%. Comprehensive monitoring programs and adaptive management strategies are vital for identifying and mitigating environmental impacts, reducing undetected impacts by up to 75% [98] and mitigating long-term irreversible impacts by up to 80% [101].

To address the risks associated with CRISPR/Cas9 in aquaculture, it is important to establish a comprehensive regulatory framework and rigorous biosafety protocols. These risks include potential escape and interbreeding with wild populations, transferring engineered traits, and altering the fitness and behavior of wild fish, potentially disrupting the balance of ecosystems and causing cascading effects throughout the food web [102]. Non-target species may also experience unpredictable ecological impacts, leading to potential biodiversity loss.

To start, implement specific containment strategies, such as biotechnological approaches like triploid induction, to produce fish that are sterile or have limited reproductive capacity. Additionally, ensure rigorous physical containment by using secure enclosures [97].

However, long-term monitoring programs that track ecological effects, including changes in population dynamics, biodiversity, and ecosystem functions must be established. Develop adaptive management strategies to respond quickly to unforeseen ecological impacts, adjusting practices based on monitoring results and emerging scientific insights.

Moreover, involve diverse stakeholders in decision-making processes, including local communities, scientists, policymakers, and industry representatives. Utilize effective communication strategies to raise public awareness about the risks and benefits of CRISPR/Cas9 technology, fostering informed decision-making and building public trust [63].

Furthermore, establish global standards and guidelines for risk assessment and regulatory oversight to ensure consistency across countries. Facilitate the transfer of CRISPR expertise and technical knowledge to research institutions and extension services in developing regions, building local capacity and promoting the responsible use of this technology.

## 12. Challenges and Limitations Genome Editing Technology in the Genetic Improvement of Fish in Aquaculture

Diseases pose significant challenges for fish farmers, impacting investment plans and increasing costs. Although CRISPR/Cas9 is a promising tool for genetic improvement in aquaculture, it carries potential risks, such as the introduction of new traits that might affect disease dynamics if GMF interbreed with wild populations. Genetic modification itself does not inherently increase disease transmission, but altered genes can have unintended effects on immune-related functions, potentially increasing disease susceptibility [103]. Concerns have been raised about genetically modified Atlantic salmon potentially spreading diseases to wild populations [104].

Aquaculture technology can impact biodiversity by modifying fish species for favorable traits, potentially outcompeting wild populations and altering ecological dynamics. This may lead to reduced genetic diversity and changes in ecological interactions. Public concerns about genetically modified organisms (GMOs) influence consumer behavior, affecting market demand and price. Additionally, ethical issues related to animal welfare and unforeseen outcomes associated with CRISPR/Cas9 in animal breeding emphasize the need for appropriate regulations and oversight.

**Figure 3:**
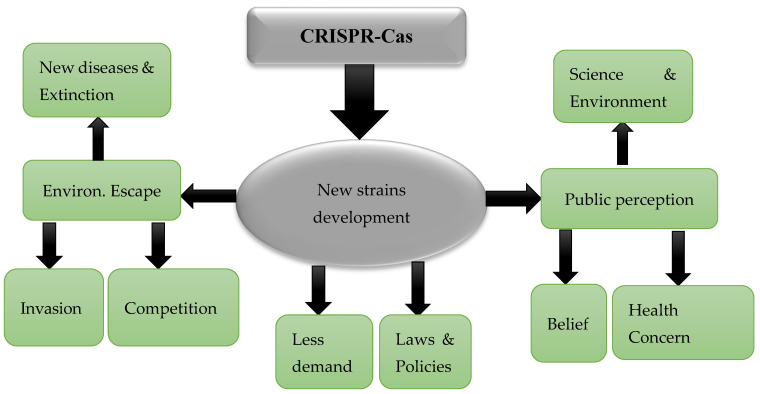
Depicts the summarized challenges of developing new strains using CRISPR/Cas9 technology. The main arrow pointing downwards represents the development process, while smaller arrows indicate the various challenges associated with accepting the new strain resulting from this technology. In Figure 3, two major challenges to the acceptability of the new strain through CRISPR/Cas9 technology are illustrated. These challenges are: (1) the potential escape of the new strain into the environment, which could lead to the invasion of indigenous strains, the introduction of new diseases, and increased competition, and (2) the concern that modified new strains may lead to certain public perceptions, such as health concerns, religious/personal beliefs, and environmental concerns. However, it is important to note that public perception towards the development of new strains is influenced by people’s beliefs, values, attitudes, needs, and interests [108]. Personal awareness of stimuli also plays a significant role in shaping attitudes towards science and the environment.

CRISPR/Cas9 technology faces challenges such as off-target effects, regulatory barriers, and public perception issues, which can affect environmental sustainability and fish health. Efforts are underway to improve specificity and reduce off-target effects. The cost and scalability of CRISPR/Cas9 applications are important considerations, alongside ongoing discussions about ethical issues and technological limitations. Although CRISPR/Cas9 has the potential to revolutionize aquaculture by editing fish genomes, potential drawbacks include reduced fertility, increased disease risk, and ecological consequences if genetically engineered fish interbreed with native populations (see Figure 3).

Many CRISPR/Cas9 applications in aquaculture remain theoretical, requiring further study to assess their risks and benefits. Challenges include limited genetic resources for aquatic species, which complicates the identification of trait-related genes. Additionally, microinjection success rates are low in egg-laying fish due to the scarcity of egg membrane material. There is no standard protocol for genome editing in aquatic organisms, with successful applications limited to species like slipper mollusks, shrimp, and oysters due to technical constraints [19,105,106].

Further refinement of CRISPR/Cas9 techniques is necessary to fully realize their potential in aquaculture. Long generation intervals in aquatic species make genome editing time-consuming, but combining it with surrogacy technology may provide a solution [107]. Producing sterile organisms is the preferred strategy for commercial applications to protect intellectual property and prevent unintended genetic invasion. Evidence suggests that germ cells in Atlantic salmon can be manipulated to produce sterile offspring, mitigating risks associated with modified fish escaping into the wild [31].

## 13. Public Acceptability and Concern for Genetically Modified Fish

Public concern plays a crucial role in the advancement of genetic modification in aquaculture. The acceptance of genetically modified fish, especially those altered using CRISPR/Cas9, is influenced by cultural, ethical, and environmental factors [109,110]. It is important to understand these concerns to successfully develop and implement genetic modification technologies in aquaculture.

Consumers prioritize safety and quality when it comes to genetically modified fish. They are concerned about potential risks, including toxicity, allergenicity, and changes in nutritional value. Ethical considerations are also significant in the genetic modification debate. Questions about the moral status of animals, the idea of naturalness, and the value of species greatly affect public perception and acceptance [111]. Furthermore, environmental impacts such as gene flow to wild populations and biodiversity changes raise significant public concerns. Studies suggest that genetically modified fish may affect disease resistance, behavior, and reproductive patterns, leading to ecological questions [112,113].

Cultural attitudes towards genetic modification differ across regions. For example, Europeans have historically been skeptical of genetically modified foods compared to other regions [114]. This skepticism is evident in the strict regulations imposed by the European Union on GMOs, requiring rigorous risk assessments and labeling to ensure safety and consumer trust.

On the other hand, the United States generally takes a more permissive approach to GMOs, including genetically modified fish. Regulatory bodies like the FDA focus on evaluating safety and environmental impact, but often face criticism for being too lenient [111]. In contrast, Japan adopts a cautious stance, taking public opinion into account and prioritizing safety and consumer preferences in its regulatory approach [109]. Canada follows a science-based framework, having approved genetically modified salmon for production and consumption while emphasizing transparency and public engagement [110].

In developing countries, policies vary widely and are often influenced by economic needs, technological capacity, and international trade considerations. Public acceptance in these regions tends to be lower, with significant concerns about environmental impacts and food security.

Despite high public skepticism due to cultural and ethical considerations, the European Union maintains strict regulations for GMOs [115]. The United States takes a more lenient stance on GMOs, prioritizing safety and consumer preferences. Japan, on the other hand, approaches GMOs with caution, also emphasizing safety and consumer preferences. According to Ishii and [109], public opinion plays a significant role in shaping Japan’s more conservative regulatory stance. In Canada, a science-based regulatory framework is utilized, with a focus on transparency and public engagement. As demonstrated by the approval of genetically modified salmon [110]. In order to enhance public acceptance of genome-editing technologies, it is important to consider cultural values and concerns, establish trust with community leaders, and ensure transparent regulatory processes and open dialogue. Education and awareness initiatives can effectively address fears and misconceptions, while ongoing monitoring of ecosystem impacts and long-term effects is crucial for maintaining public confidence. By implementing these measures, we can foster a more informed and supportive public perspective on genome-editing technologies.

The acceptance of genetically modified fish by the public is a complex and ever-evolving issue. Cultural, ethical, and environmental factors must be carefully considered. The differences in public opinion and policy among countries underscore the importance of tailored regulatory approaches and effective communication strategies. As technologies like CRISPR/Cas9 continue to advance, ongoing dialogue and transparent governance will be essential for addressing public concerns and promoting acceptance. The journey towards wider acceptance of fish modified using CRISPR/Cas9 technology should involve the active participation of the public, stakeholders, and experts in a constructive and inclusive manner, as summarized in Table 3.

## 14. Potential Solution to Public Perceptions and Concerns Regarding the Challenges of Genome Editing Fish

The application of genome editing technologies, especially CRISPR/Cas9, in aquaculture presents both opportunities and challenges. These technologies have the potential to improve growth rates, disease resistance, and environmental adaptability in fish, thereby enhancing aquaculture productivity and sustainability [119]. However, releasing genetically modified fish into the wild raises significant environmental and ethical concerns, requiring comprehensive environmental risk assessments [120]. 

Thorough environmental risk assessments are essential before introducing genetically modified fish into natural ecosystems. These assessments aim to identify and mitigate potential ecological risks, such as effects on biodiversity and ecosystem stability. By addressing these concerns, researchers and stakeholders can ensure that the use of genome-edited fish aligns with sustainable aquaculture practices [121] (see Table 4).

Effective governance relies on collaboration among academia, industry, regulatory agencies, and policymakers to establish comprehensive regulations that promote responsible use of CRISPR/Cas9 technology in aquaculture. These frameworks must ensure that ethical and safety standards are met while maximizing the benefits of genetic enhancements [122]. Clear guidelines and transparent communication with the public are essential for building trust and addressing societal concerns [123].

Monitoring genetically modified fish populations is vital to evaluate their long-term impact on ecosystems and aquaculture sustainability. Developing robust monitoring methods can help detect unexpected effects and facilitate adaptive management strategies [55]. This approach ensures that any adverse outcomes can be addressed promptly, maintaining ecological balance and public confidence in genome editing technologies.

In addition to CRISPR/Cas9, other gene editing techniques like base editing and prime editing offer precise genomic modifications. These technologies provide additional tools for achieving targeted genetic improvements in aquaculture species, broadening the scope of potential enhancements [123,124]. Continued research and development in these areas can diversify the genetic strategies available for sustainable aquaculture.

Ensuring fair access to CRISPR/Cas9 technology is crucial for fostering innovation and preventing monopolization. Open-source platforms, collaborative research initiatives, and affordable licensing models can facilitate broader utilization of genome editing tools among researchers and industry stakeholders [125]. By promoting inclusivity and collaboration, the aquaculture sector can maximize the potential of genome editing to address global food security challenges.

**Table 4:** Advancements and Safety Measures in CRISPR/Cas9 Gene editing for Aquatic species.

Remedies	Impact	Ref.
Improved precision	It improves the precision of developing guide RNA molecules that are more efficient and accurate, minimizing off-target effects and enhancing gene editing specificity.	[126]
Enhanced delivery methods	It enables scientists to develop new delivery methods, such as electroporation, microinjection, or transfection, to improve the efficiency of delivering CRISPR/Cas9 components into fish embryos for gene editing in aquaculture.	[127]
Gene drive systems	CRISPR/Cas9 helps researchers to gene drive systems to quickly introduce advantageous traits, such as disease resistance or enhanced growth, into a fish population.	[44,128]
Biosecurity measures	Secure laboratories and greenhouses are utilized to contain and minimize the risk of accidental release of GMOs into the environment.	[119]
Public engagement and education	Educating the public improves understanding of CRISPR/Cas9, which in turn enables better comprehension of its mechanisms, benefits, and limitations in aquaculture and other applications.	[102]
Targeting complex traits	CRISPR/Cas9 allows for the simultaneous targeting and modification of multiple genes or genetic loci in fish species.	[129]
Ethical considerations	The ethical discussions regarding gene editing in aquaculture are important, as they consider animal welfare, environmental impact, and potential unintended consequences.	[116,124]

## 15. Ethical Considerations of the Use of CRISPR Technology in Aquaculture and Its Impact on Food Security

The emergence of CRISPR/Cas9 technology has revolutionized various fields, including aquaculture, by providing the potential for precise genetic modifications in aquatic species. These modifications offer the promise of improved growth rates, disease resistance, and overall productivity. However, the use of CRISPR/Cas9 in aquaculture presents several ethical and practical concerns that need to be addressed to fully maximize its benefits while minimizing potential drawbacks.

One major ethical concern regarding CRISPR-based aquaculture technologies is ensuring equitable access, especially for small-scale and marginalized producers in developing countries. There is a risk that advanced technologies like CRISPR may predominantly be accessible to large-scale producers in wealthier nations, potentially worsening existing disparities in the aquaculture sector [130,131]. This could result in concentrated benefits for the few, leaving vulnerable populations without access to the potential productivity gains offered by these innovations. To address this issue, strategies need to be developed to ensure that small-scale and marginalized producers can also benefit from CRISPR-based advancements [77,132].

Ecological concerns also arise with the introduction of CRISPR-modified organisms. There is a risk of unintended escape and proliferation of genetically modified species, which could disrupt native ecosystems and lead to unforeseen long-term environmental impacts [86,133]. Therefore, careful risk assessment and stringent regulation are necessary to mitigate these potential environmental consequences [44,95].

Public resistance to consuming CRISPR-derived seafood is another significant consideration. In regions where genetically modified organisms face skepticism, it is essential to prioritize transparency, clear labeling, and addressing consumer concerns about food safety and ethics to gain public acceptance [22,132]. Effective communication and addressing ethical concerns will play a crucial role in facilitating the adoption of CRISPR-edited aquaculture products.

The introduction of CRISPR-based aquaculture also has sociocultural implications. It has the potential to disrupt traditional fishing and aquaculture practices, alter local food systems, and possibly undermine the livelihoods and food security of small-scale producers [134,135]. Therefore, careful consideration needs to be given to the sociocultural impact of integrating genetic technologies into traditional practices to avoid any adverse effects on local communities [136,137].

CRISPR-based aquaculture has the potential to increase food security by improving productivity and reducing costs of fish and seafood. This could make them more affordable and accessible for low-income consumers in developing regions [90,131]. It would also enhance food and nutrition security by increasing the availability of affordable, nutrient-rich animal protein. However, introducing CRISPR-modified products into global trade could disrupt existing markets and supply chains. This could lead to trade barriers and uneven distribution of benefits [130,131]. To address these risks and concerns, inclusive governance frameworks are needed. These frameworks should involve diverse stakeholders, including small-scale producers, local communities, and consumers, in the development and regulation of CRISPR-based aquaculture technologies [134,138,139].

In conclusion, while CRISPR-based aquaculture offers opportunities for enhancing productivity and food security, it requires a balanced approach that considers ethical, ecological, and sociocultural challenges. Steps such as ensuring equitable access, mitigating environmental impacts, addressing public concerns, and considering sociocultural implications are essential to realize the full potential of CRISPR-based aquaculture technologies while protecting the interests of all stakeholders involved.

## 16. Conclusions

The advancement of genetic improvement in fish species is crucial for the evolution of aquaculture, especially in rapidly expanding regions like Asia. CRISPR/Cas9 technology has emerged as a transformative tool in this field, allowing precise modifications to enhance desirable traits such as disease resistance, growth rates, and feed conversion efficiency. This gene-editing technology has the potential to revolutionize aquaculture by providing solutions to long-standing challenges in disease management and genetic optimization.

However, the application of CRISPR/Cas9 in aquaculture also raises significant concerns. Ecological impacts, such as the potential escape of genetically modified fish into natural habitats and their effect on native species, as well as social and ethical considerations regarding public perception and acceptance, need to be addressed.

To maximize the potential of CRISPR/Cas9 technology while mitigating associated risks, future research should focus on several crucial areas. One key direction is the development of advanced gene-editing techniques that ensure greater precision and minimize off-target effects. Improvements in delivery methods, such as enhanced vectors and less invasive procedures, could enhance the efficiency and safety of gene editing in aquatic species.

Additionally, long-term studies are essential to assess the ecological impacts of CRISPR-modified fish on natural ecosystems. Comprehensive risk assessments and monitoring programs will be crucial for understanding and managing the environmental implications. Public acceptance and regulatory frameworks must adapt to address consumer concerns and establish clear guidelines for the responsible use of gene-edited organisms.

Furthermore, future research should explore the integration of CRISPR/Cas9 technology into sustainable aquaculture practices. This involves developing strategies for the responsible management of genetically modified fish in both controlled environments and open systems. Advances in genomics and biotechnology could enable the creation of fish strains that are not only optimized for growth and disease resistance but also contribute to the overall sustainability of aquaculture operations.

In conclusion, CRISPR/Cas9 technology has immense potential to advance the aquaculture industry by improving fish genetics and promoting more sustainable practices. By addressing current challenges and focusing on forward-looking research, the aquaculture sector can harness the full capabilities of gene editing technologies to build a more resilient, efficient, and environmentally responsible industry. Collaboration among researchers, regulators, and stakeholders will be crucial in realizing the long-term benefits of CRISPR/Cas9 technology in aquaculture as we navigate these developments.

## Figures and Tables

**Table 1:** Effects of CRISPR/Cas9 on Biological and Environmental Aspects of Fish Species.

Applicable Fields	Impacts	Ref.
Disease resistance	It is used to reduce the viral hemorrhagic septicemia virus (VHSV) infection of olive flounder hirame natural embryo (HINAE) cells.	[84]
	It enables gene editing in fish species such as salmon, tilapia, and shrimp to increase their resistance to diseases.	[85,86]
	It helps in the deletion of the *JAM-A* gene in grass carp cells, which significantly enhances resistance to grass carp reovirus (GCRV) infection.	[39]
	It helps enhance fish cell lines for host response and genetic resistance against infectious diseases, using Atlantic salmon and rainbow trout as model systems in aquaculture.	[22]
Environmental adaptation	It helps to edit genes in fish species, such as farmed salmon, to adapt to changing environments.	[31,86]
Improved growth rates and muscles	It increases muscle growth by knocking out melanocortin (*mc4r*) receptor genes and has been experimentally tried on channel catfish and medaka fish.	[28,87]
	It improved the growth rates and increased muscle mass of the channel catfish by modifying the myostatin gene in channel catfish embryos.	[12]
	It helps increase the muscle mass of blunt snout bream due to the disruption of the *mstna* and *mstnb* genes.	[48]
Bone development	It helps in *myostatin* gene disruption of genes, such as transcription factor *sp7*, causing bone defects in common carp, and increases muscular cells, resulting in a more robust muscular phenotype.	[46]
Colour defects	It can be used to edit genes involved in pigmentation pathways, potentially leading to loss of skin pigmentation, e.g., edited mutant of large-scale loach, causing skin pigmentation loss and black patch dispersion in the Oujiang color common carp.	[19,68]
	It helps identify and introduce mutations in genes responsible for pigmentation, such as *tyrosinase* or *mitf*, which can lead to pigmentation defects in fish species like salmon.	[68]
	It helps to reveal a recessive inheritance pattern for the white-albino phenotype, lacking pigment-containing chromatophores, in rainbow trout.	[88]
Sex determination	It can be used to disrupt or modify the gonadal soma-derived factor (*gsdf*) gene, which is a crucial gene in teleost fish. Disruption of genes such as dmrt1 and cyp19a1a can lead to sex reversal phenotypes in zebrafish.	[89]
DNA integration	It facilitates the integration of exogenous DNA into the zebrafish genome, but it may also cause additional genetic mutations or disruptions, depending on the editing conditions and precision of the technique.	[90]
kidney and gonads development	It helps disrupt the *Wilms tumor 1* (*wt1a*) gene, which may lead to abnormal gonad and kidney development in Nile tilapia.	[69]
Immune genes improvement	It has been used to knock out or edit genes in salmon fish. However, overexpressing interferon (*IFN*) or inducing stimulated genes (*ISGs*) does not guarantee broad disease resistance.	[36]

**Table 2:** Applications of CRISPR/Cas9 in Various Fish Species and Their Impacts.

Fish Species	Technological Impacts	Ref.
*Nile tilapia*	It is used to produce sterile Nile tilapia populations, reducing the risk of environmental damage from escaped fish.	[11]
*Atlantic salmon*	It helps in gene editing to create species that are highly resistant to viral infections, e.g., salmon.	[18]
*Zebrafish*	It allows scientists to study mutations and genetic variants in zebrafish.	[93]
	It can be used to successfully integrate composite tags into zebrafish embryos, enabling precise labeling and visualization of cellular structures or proteins. This offers potential for studying protein dynamics, gene expression, and other biological processes in this model organism.	
*Rainbow trout*	It has been shown to reduce the expression of the *igfbp-2b* gene in rainbow trout, influencing growth and development, but its impact on overall performance and the endocrine system remains unclear.	[35]
*Atlantic salmon and Rainbow trout*	It has been used to target unique genes associated with growth and immunity in Atlantic salmon, rainbow trout, and coho salmon cells.	[22]
*Japanese medaka*	It has the potential to increase muscle growth and body weight in farmed fish species such as medaka. However, further investigation is needed to determine its impact on production yield and fish health.	[28]
*Olive flounder*	It can be used to disrupt the myostatin gene in olive flounder, potentially increasing body weight and muscle tissue, but further research is needed to understand its effects on production efficiency and fish health.	[49]
*Channel catfish*	It has been used to modify the *myostatin* gene in Channel catfish to improve muscle growth and quality, but further research is needed to fully understand its effects.	[12]

**Table 3:** Key Factors Influencing Public Perception and Acceptance of Genetically Modified Fish.

Public Concerns	Impacts	Ref.
Awareness and education	It helps to increase public understanding and awareness of GMOs, particularly CRISPR-modified fish, and can significantly impact their acceptance and safety.	[10,102]
Benefits and risks	It improved nutrition and reduced environmental impact. Emphasizing safety assessments and risk mitigation strategies can significantly influence public opinion.	[102,116]
Ethical and environmental considerations	Public acceptance of genetically modified fish may be influenced by ethical and environmental concerns. Clear communication, transparency, and engagement play an essential role in gaining public trust.	[116,117]
Transparency and engagement	Public participation, achieved through an open dialogue among various stakeholders such as consumers, scientists, policymakers, and environmental organizations, will foster trust and acceptance.	[102]
Food labeling and consumer choice	Clear labeling and transparent information about modified fish products can assist consumers in making informed decisions regarding food consumption and safety.	[116,118]
Environmental sustainability	It provides potential environmental and sustainable aquaculture benefits, such as reduced antibiotic use and improved resource efficiency, which could have a positive impact on public acceptance.	[102]
